# Biocide-Containing Facades Alter Culture-Based Bacterial and Fungal Community Composition and Resistance Patterns to Octylisothiazolinone

**DOI:** 10.3390/microorganisms13102284

**Published:** 2025-09-30

**Authors:** Michał Ciok, Julia Diener, Franziska Otte, Julie Feimer, Moritz Nichterlein, Stefan Kalkhof, Matthias Noll

**Affiliations:** 1 Institute of Bioanalysis, Coburg University of Applied Sciences and Arts, 96450 Coburg, Germanystefan.kalkhof@hs-coburg.de (S.K.); 2 Proteomics Unit, Fraunhofer Institute for Cell Therapy and Immunology, 04103 Leipzig, Germany

**Keywords:** façade, discoloration, biodeterioration, culture-based microbial community composition, minimal inhibitory concentration (MIC), octylisothiazolinone, biocide resistance, biofilm adaptation

## Abstract

Microbial communities are known to colonize biocide-free (BFFs) and even biocide-containing façades (BCFs) under various environmental conditions, leading to loss of value of façades due to biologically caused aging and discoloration. The first objective of this study was to characterize the bacterial and fungal cultivation-based communities present on BCFs and BFFs after one year of outdoor exposure. The second objective was to assess their tolerance to biocide octylisothiazolinone (OIT), which was only present on the BCFs. Culture-based analysis revealed significant differences in bacterial community composition between the BFFs and BCFs. Fungal isolates also varied, with *Penicillium* predominantly found on the BCFs and *Vishniacozyma* and *Memnoniella* on the BFFs. MIC testing showed that the isolates from the BCFs exhibited slightly higher tolerance to OIT than those from the BFFs, although the differences were not statistically significant. Notably, several bacterial genera identified in both façade types—*Clavibacter*, *Micrococcus*, *Nocardioides*, *Rhodococcus*, and *Streptomyces*—as well as the fungal genus *Penicillium*, have previously been reported to degrade biocides. These findings demonstrate that both BF and BC façades support taxonomically diverse and resilient microbial communities within a relatively short exposure period. While minor shifts in biocide tolerance were observed, the lack of significant differences suggests that microbial adaptation to biocide-containing façades may be more complex and gradual, underscoring the need for time-resolved and functional studies to better understand microbial adaptation to biocide in façades.

## 1. Introduction

Façades are constantly exposed to multiple environmental and weathering influences [1,2], as well as a diverse airborne microbial community capable of colonizing surfaces. Microbial colonization is governed by both microscale and macroscale environmental parameters. Microscale factors include humidity, light, render composition (e.g., salinity and pH), and render structure (e.g., roughness and porosity). Macroscale factors comprise air temperature, UV radiation, and climate conditions (e.g., wet and dry seasons), as well as the concentration and types of biogenic and anthropogenic airborne particles (e.g., dust, pollen, and other colonizers) [3,4,5]. Depending on the presence and extent of microscale and macroscale factors, microbial façade colonization steadily increases over time by bacteria, fungi, algae, lichens, and mosses [3]. This colonization contributes to an ever-increasing biodeterioration of the façades. Biodeterioration entails the adverse modification of the physical features of façades that are often caused by microbial colonization [6]. While the roles of fungi and algae in façade biodeterioration have been extensively studied, bacterial contributions remain underestimated, especially in German climate conditions [3]. Recent research on residential facades (5–21 years old in Portugal) revealed diverse bacterial communities, mainly from the phyla Cyanobacteria, Proteobacteria, Actinobacteria, Bacteroidetes, as well as fungi such as *Cladosporium* and *Penicillium* [7]. However, a previous study used a cultivation-independent approach without collecting live microorganisms for further investigations, including biochemical characterization and resistance testing against façade-incorporated biocides [7]. A cultivation-dependent technique enables the isolation of cultivable strains, facilitating phenotypic analysis, biochemical profiling, and biocidal susceptibility testing by assessing the minimum inhibitory concentrations (MICs) [8]. In this study, this approach was chosen specifically to establish a cryo-preserved culture collection from the BFFs and BCFs. This living database not only allowed us to investigate MIC values and biochemical traits under defined exposure conditions, but it also provides a unique resource for future research on façade-associated microorganisms. The aesthetic biodeterioration of façades encircles multiple factors, including discoloration, surface damage, and the accumulation of environmental dirt. However, this study specifically defines discoloration as an indicator of microbial colonization. This phenomenon primarily involves organisms from the phylum Cyanobacteria, kingdom fungi, and/or algae, neglecting the role of bacteria [9,10,11], which support, interact, and/or counteract with each other, thereby affecting façade colonization patterns.

The producers of façade compounds (e.g., render and paint) have different strategies to overcome the occurrence and extent of microbial biodeterioration and discoloration. Some manufacturers use water-repellent or water-absorbing materials without biocides or photocatalytic active agents for self-cleaning façades. Nevertheless, in-can and dry-film preservatives are still commonly applied to prevent microbial growth [12,13,14,15,16]. In Europe, such biocides are regulated by Biocide Products Regulation (BPR) (EU) No. 528/2012, which covers their use in building materials and coatings [17,18]. Biocides in façade compounds can serve two main functions [14]. Biocides in façade compounds can also be used for both in-can preservatives to prevent microbial growth during storage [19,20] and film preservatives to avoid microbial growth directly on the façade surface after application [21,22]. In-can preservatives are mostly represented by a single biocide or a mixture of biocides from the isothiazolinone group. These include benzisothiazolinone (BIT), methylisothiazolinone (MIT), and chloromethylisothiazolone (CMIT) [23]. In turn, film preservatives always include a mixture of biocides from different chemical groups, such as isothiazolinones, triazines, and/or pyrithiones [14,17]. However, the European Commission has a specific concentration limit of 15 ppm for classification as a skin sensitizer, while higher concentrations require appropriate hazard labeling [24]. Zinc pyrithione has been banned from cosmetic products in the EU since 2022 following its classification as a reproductive toxicant. However, its use in coating materials remains hazardous, where there are labeling requirements when under BPR with >100 ppm. The isothiazolinone group is known for its bacteriostatic and fungistatic characteristics. These are achieved through interactions with active proteins (thiol groups in cysteine), which inhibit enzyme activity and lead to cell disintegration [25]. When applied to façades, these antimicrobials are used at concentrations significantly higher than the individual MICs of the target (micro)organisms to ensure their long-term effectiveness. However, MIC testing and standard formulations are generally optimized under laboratory conditions and do not account for other environmental factors. Therefore, outdoor façade exposure studies and targeted sampling initiatives are necessary to assess the actual microbial colonizers on facades and their current biocide susceptibilities.

Most published studies have applied comparable biocide mixtures and concentrations as a model for experimental façades, including the film preservative OIT [1,19]. In contrast to in-can preservatives, which are typically leached out rapidly after application [1,19], film preservatives such as OIT exhibit a prolonged presence over several years regarding their release and degradation kinetics rates [26,27]. To enhance long-term availability and biocidal effectiveness under outdoor conditions, biocides have been used in an encapsulated product for the last 15 years [16]. This technology shields biocide molecules within polymeric micro- and nanocarriers, safeguarding them against environmental factors [28]. The approach of encapsulation slows down the release of biocides from the façade, thereby mitigating the leaching behavior induced by wind-driven rain, moisture, UV radiation, and other weathering factors [29]. Additionally, microbial pigment distribution contributes to façade discoloration and may serve as a proxy for radiation survival. Likewise, catalase and oxidase activity indicate a phenotype capable of coping with reactive oxygen species, particularly on biocide-treated surfaces. Finally, Gram differentiation can be useful, as Gram-negative bacteria have been described as more resistant to environmental stressors [30].

To the best of our knowledge, a cultivation-dependent approach of bacterial and fungal strains from biocide-containing (BCFs) versus biocide-free (BFFs) façades, along with their phenotypic and biochemical characterization, is thus far missing. Such data are crucial for assessing whether and to what extent current biocide applications can be altered by bacteria or fungi and potentially deactivated. Finally, the strain collection can be employed to determine the MICs of OIT applied in BCFs and could be used in the future for innovative and more realistic approaches to biological façade testing methods.

## 2. Materials and Methods

### 2.1. Description of the Field Trial

The field trial was carried out as has been described previously [19]. Briefly, biocide-containing (BCFs) and biocide-free (BFFs) façades were applied to three L-shaped concrete blocks measuring 0.4 × 0.4 m at the University of Applied Sciences Coburg, Germany (coordinates 50°15′ N, 10°56′ E, AMSL-331 m). The formulation for the acrylic façade materials (render and paint) has been previously published and adopted with minor modifications [1]. These modifications include the replacement of Acticide MBS 5050 by Acticide SR 2081 (both Thor GmbH, Speyer, Germany), as well as Acticide MKB 3 (Thor GmbH) with concentrations of 350 mg * kg^−1^ for renders and 500 mg * kg^−1^ for paints, respectively. Additionally, DPnB (1%) by the same amount of water was included [19]. The BCFs contained 2-Methyl-4-isothiazolinone-3-one (MIT) (Thor GmbH, Speyer, Germany): 7 mg * kg^−1^ dry weight (dw); Methylchloroisothiazolinone (CMIT) (Thor): 20 mg * kg^−1^ dw; 1,2-Benzisothiazol-3(2H)-one (BIT) (Thor): 395 mg * kg^−1^ dw; terbutryn (TER) (Thor): 173 mg * kg^−1^ dw; OIT: 97 mg * kg^−1^; and zinc pyrithione (ZnP) (Thor): concentrations of which could not be detected in the building facades using standardized protocols after outdoor exposition. All materials of the BFFs were classified by the respective manufacturer as biocide-free. However, minor residues of BIT were detected in the BFF eluate (0.05 mg/L) and were classified as non-toxic based on toxicity test results on bacteria and other organisms [19]. These may be attributed to undisclosed in-can preservatives or they were carried over from the preservative systems used in raw materials such as latex dispersions or pigment fillers [31].

**Figure 1:**
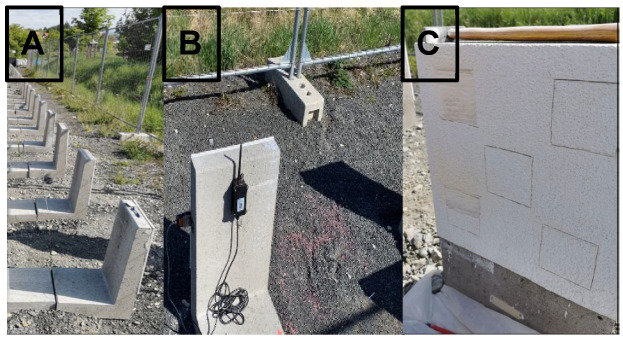
Field trial façades (**A**) with attached weathering sensors (**B**) and sampling approach (**C**) from the BCFs and BFFs, respectively. Three independent BCFs and BFFs, which all faced the south/east orientation, were sampled five times (see 0.08 × 0.08 m squares in the plaster for analysis).

The field trial was performed from April 2021 to May 2022, covering a full year of exposure and thus all weathering seasons. Meteorological data (temperature and moisture) were recorded during the summer season, from May to August 2021 (see Figure 1B). Microorganisms were directly isolated from the BFFs and BCFs in May 2022 without prior storage, followed by biochemical and sequencing-based characterization and MIC susceptibility testing. Sampling was carried out in three independent replicates of the BCFs and BFFs, each with five sub-replicates (in total, 30 samples and 3 × two treatments [BCFs, BFFs] × 5 sub-replicates, see Figure 1C), all with the same south-east side (S/E) orientation. This orientation was opposite the weathering site (see Figure 1A). The average humidity percentages for the BFFs and BCFs were comparable (BFFs: 9.3% mean, 2.2% min, and 16.4% max; BCFs: 9.4% mean, 2.1% min, and 17.5% max). Similarly, the temperature data were also comparable between the BFFs and BCFs (BFFs: 18.8 °C mean, 1.5 °C min, and 43.5 °C max; BCFs: 19.0 °C mean, 1.6 °C min, and 44.0 °C max) [19]. These meteorological data were recorded to ensure the statistical insignificance between the BFFs and BCFs and were not subjected to statistical correlation with microbial abundance or resistance.

### 2.2. Sampling

The BCFs and BFFs were sampled using a multifunctional grinding tool (Parkside PFBS 12 B3, Bochum, Germany) from five 0.08 × 0.08 m sampling squares of each façade: top right, top left, middle, bottom right, and bottom left (see Figure 1C). Each of the 4 stripes (0.08 m × 0.02 m) resulted in approx. 16 cm^2^ and 4-to-5 g plaster strips, which were collected in sterile Petri dishes (Greiner Bio-One GmbH, Frickenhausen, Germany). This method was chosen because it ensured a defined sampling area and allowed for the collection of microorganisms from both the surface and subsurface layers of the material. All stripes of one sample were separately ground using an autoclaved mortar and pestle (Greiner Bio-One) and sieved to a particle size of <0.4 mm.

### 2.3. Isolation and Cultivation of Bacterial and Fungal Façade Colonizers

The culture-based protocol to retrieve bacteria and fungi from the BCFs and BFFs was adapted from established methods [7,32,33], with minor modifications in the cultivation approach for fungi. Specifically, potato glucose agar (PGA; dextrose substituted with glucose) (Carl Roth GmbH + Co. KG, Karlsruhe, Germany) was used instead of the commonly applied potato dextrose agar (PDA), as PGA has shown comparable performance for façade isolates [34,35]. Briefly, 1 g of pulverized façade from each sample was resuspended in a ¼ Ringer solution (Merck KGaA, Darmstadt, Germany) (38.5 mM of NaCl, 1.408 mM of KCl, 0.54 mM of CaCl_2_, and 0.596 mM of NaHCO_3_ at pH 7) and was two times 10-fold diluted and thereafter mixed by vortex mixer. Afterward, 100 µL of the 1:1; 1:10, and 1:100 suspensions were plated on Mueller–Hinton agar (MHA) (Carl Roth GmbH + Co. KG, Karlsruhe, Germany). The MHA plates were incubated at 30 °C in darkness for both façade types (BCFs and BFFs). In addition, 100 µL of the 1:1; 1:10, and 1:100 suspensions were plated on PGA, which was also used for the subsequent susceptibility testing of fungi [34]. PGA plates were incubated at 25 °C in darkness, and the MHA, as well as PGA, plates were optically inspected every 48 h for two weeks [34]. An MHA medium was chosen to isolate strains that could be directly used for antimicrobial susceptibility testing, as these tests were conducted using the same MHA according to EUCAST guidelines. MHA was chosen because it supports a broad range of non-fastidious bacteria and allows direct use of isolates for antimicrobial susceptibility testing following EUCAST guidelines [36]. The PGA supported fungal growth and was used for subsequent antifungal susceptibility testing, as has been previously described with the minor modification noted above [34]. Only single colonies from the 1:1 suspension were picked for subsequent separation, as well as the same media and incubation conditions to be obtained after several purification steps to pure cultures [33]. Due to space limitations on the original agar plates and the simultaneous growth of multiple colonies, not all colonies could be individually isolated and purified into pure cultures. However, we avoided any pre-selection based on pigmentation or other colony morphology types. Moreover, we applied standardized sampling, plating, and cultivation procedures to optimize the reproducibility of our culture-based approach. Colony color, shape, texture, size, and presence of visible hyphae or spores from each strain were documented. To distinguish between Gram-positive and Gram-negative strains, a 3% KOH solution was applied [37]. The presence of the catalase enzyme was analyzed as has been outlined earlier in an established and adopted protocol [38]. To evaluate the oxidase activity of the collected stains, oxidase test stripes from Merck KGaA were used in accordance with the manufacturer’s guidelines. Pigment colorization was standardized and recorded as has been introduced in previous studies [39,40].

### 2.4. DNA Extraction and Amplification of Isolates from Façades

DNA was extracted as described earlier [41], albeit with major modifications. Briefly, a colony of each isolate was individually picked with a sterilized inoculation loop and transferred into 500 µL of ¼ Ringer solution. The cell suspensions were vortexed for 30 s, transferred into 500 µL of Roti^®^-Chloroform-Isoamylalkohol (Carl Roth GmbH + Co. KG), and then centrifuged for 5 min at 14,000× *g* at 4 °C. Subsequently, 350 µL of the supernatant was transferred into new reaction tubes filled with 700 µL of a 30% Polyethylenglycol-6000 (PEG, Carl Roth GmbH + Co. KG) and 2 µL of glycogen (Thermo Fisher Scientific, Waltham, MA, USA). Samples were incubated for 2.25 h at room temperature and thereafter centrifuged at 14,000× *g* at 4 °C for 10 min. The supernatant was discarded, and the nucleic acid pellet was washed twice with 70% ice-cold ethanol (Carl Roth GmbH + Co. KG). For DNA drying, the washed samples were placed in a Vacuum concentrator (Speed Vac, Eppendorf, Hamburg, Germany) at 2213× *g*, for 15 min, and at 30 °C. The pellet was resolved in 50 µL of TPM buffer (50 mM Tris-HCl; 20 mM MgCl_2_; 1,7% (*w*/*v*) Polyvinylpyrolidon K25; pH 6.8, (Carl Roth GmbH + Co. KG) and stored in a refrigerator at −20 °C.

For Sanger sequencing, the extracted DNA of each isolated strain was amplified by PCR using the primer set S-D-Bac-0008 (5′-AGAGTTGATCMTGGC) (Merck KGaA) and Bac1492r_R (5′-GGYTACCTTGTTACGACTT) (Merck KGaA) for bacteria and the primer set primer TS1ngs_F (5′-TCCGTAGGTGAACCTGC) (Merck KGaA) and the reversed primer ITS4ngs_R (5′-TCCTSCGCTTATTGATATGC) (Merck KGaA) for fungi. For this purpose, 20 µL of Master Mix for PCR (2-fold concentrated; Bio-Rad Laboratories Inc., Hercules, CA, USA) was used, containing 0.075 units per 1 µL of Taq DNA polymerase (Bio-Rad Laboratories Inc.) and reaction buffer (Bio-Rad Laboratories Inc.); 4 mM of MgCl_2_; 0.4 mM of each dNTP; 2.4 µL of both the forward and reverse primers, each with the concentration of 5 mM for a final concentration of 0.3 µM per assay along with 13.2 µL nuclease-free water (Bio-Rad Laboratories Inc.); and 2 µL of undiluted DNA template. As a negative control, 2 µL of nuclease-free water was added instead of a DNA template. The PCR for bacterial templates was performed in the ThermoCycler T100 (Bio-Rad Laboratories Inc.), at 94 °C for 2 min (initial denaturation), 34 cycles of denaturation at 94 °C (30 s), annealing at 48 °C (45 s), extension at 72 °C (60 s), and a final extension at 72 °C for 8 min. Similarly, the thermoprofile for fungal templates was 94 °C for 3 min (initial denaturation), 34 cycles of denaturation at 94 °C (45 s), annealing at 51 °C (45 s), extension at 72 °C (45 s), and a final extension at 72 °C for 7 min. To qualify the amount of DNA in the amplified PCR products, 5 µL of DNA template and 1 µL of 6-fold concentrated loading dye ROTI^®^Load DNA (Carl Roth GmbH + Co. KG) was run on 1% (*w*/*v*) agarose gel. Expected bacterial amplicon length was approx. 1500 bp and fungal amplicon length was up to 850 bp.

### 2.5. Sanger Sequencing and Taxonomic Assignment

Sanger sequencing was carried out by an external provider (LGC Genomics GmbH, Berlin, Germany) using Genetic Analyzer 3730XL (Thermo Fisher Scientific, Waltham, MA, USA) with POP7 polymer for electrophoresis and BigDye Version 3.1 (Thermo Fisher Scientific Waltham) in accordance with the manufacturer’s protocol. Resulting sequence data were aligned with the BLAST^®^ database. A threshold of 97% sequence identity and a query coverage of 79% was set to assign the respective taxon. Bacterial isolates, including *Staphylococcus* sp., were detected on both the BFFs and BCFs; however, based on previous studies [42,43,44], it was excluded from further analysis due to its classification as potentially human-associated, which may occasionally be deposited on façades via airborne particles or dust (though most commonly in indoor conditions [45]). Therefore, these isolates were regarded as contamination rather than as a true façade colonizer. The raw 16S rRNA gene and ITS gene sequences were deposited at the National Center for Biotechnology Information (NCBI) under the primary accession number PRJNA1148397.

### 2.6. Susceptibility Testing

Susceptibility testing has only been performed on OIT with >95% purity (HPLC) (LGC Standards, Teddington, UK) with MHB or PDB as solvent and 40 mg * L^−1^ of OIT final concentration; thus, the in-can preservatives MIT, CMIT, and BIT were rapidly leached from the façade matrix in this field study [19]. Additionally, OIT and TER were included in the BCFs as film preservatives, encapsulated for long-term protection. We focused on OIT for MIC testing due to its bacteriostatic and fungistatic properties [25], while TER primarily targets microorganisms with photosystem I and/or II. However, TER was not included in the susceptibility testing because the cultivation was conducted in darkness, thereby excluding phototrophic microorganisms. Bacterial isolates were cultured overnight in Müller–Hinton broth (MHB) at 30 °C in darkness. Bacterial isolates were cultivated until they reached a McFarland value of 1, as determined by McFarland Densitometer DEN-1B (Grant Instruments Ltd., Shepreth, UK). For subsequent susceptibility testing, each strain was diluted 100 times with ¼ Ringer solution, which refers to a final concentration of approx. 2 × 10^6^ cells * mL^−1^. The method described by EUCAST [46] with minor modifications was applied for susceptibility testing of the mold isolates. Fungal spores of each isolate were collected by adding 5 mL of a 0.9% NaCl solution to the Petri dish, and the resulting spore solution was sterile-filtered using a 2 µm PTFE filter (Rotilabo^®^, Carl Roth GmbH + Co. KG), as has been explained earlier [34]. Subsequently, the spores were counted in a hemocytometer under a microscope (Carl Zeiss Microscopy GmbH, Göttingen, Germany) at 20-fold magnification and diluted until the spore suspension reached 2 × 10^6^ spores * mL^−1^, as has been outlined in previous studies [34].

The susceptibility of bacterial and fungal isolates to OIT was tested using a 96-well plate (F-bottom; Greiner Bio-One GmbH). The final OIT concentrations ranged for the bacterial isolates from 0.25–10 mg * L^−1^ and fungal isolates from 0.125–5 mg * L^−1^. For each well, 100 µL of cell suspension was added to 100 µL double-concentrated OIT, which was prior added to MHB for bacteria or PDB for fungi. Each biocide concentration per isolate was run in four independent replicates at 30 °C for bacterial and 25 °C for fungal isolates. Additionally, each plate included a sterile control (200 µL medium only), a growth control (100 µL medium + 100 µL cells suspension without OIT), and a blank value (without medium and cells) as optical reference. The absorbance was measured at an optical density (OD) of 600 nm by a microplate spectrophotometer (BMG Labtech GmbH, Ortenberg, Germany) initially every 24 h. Initial measurements of our reference species (*Frigoribacterium faeni*, DSM 10309), one bacterial isolate (B + 10), and one fungal isolate (B + 42) were carried out in four independent overnight cultures. The cell concentration was then diluted to determine at which ΔOD_600_ growth could be distinguished. At ΔOD_600_ < 0.09, the variance of blank values was high, while at ΔOD_600_ < 0.180, the lowest OD was observed with medium and initial cell concentration. At ΔOD_600_ ≥ 0.206, growth and no-growth could be clearly distinguished for all three species. Therefore, ΔOD_600_ ≥ 0.206 was applied as the threshold for all measurements.

### 2.7. Statistics

To compare the presence, taxonomic composition, and biochemical characteristics (Gram, catalase, and oxidase), Pearson’s Chi-squared test with Yates’ continuity correction and Fisher’s exact test were conducted at a significance level of 0.05. The abundance of BCF and BFF isolates, a Shapiro–Wilk normality test, and Wilcoxon rank sum test with continuity correction was performed, with a significance level of 0.05. For MIC measurements, if at least three of the four technical replicates were detected below the limit of ΔOD_600_ ≥ 0.206, the biocide concentration was considered the MIC value. The comparison of MIC values was conducted using the Shapiro–Wilk normality test and Welch two-sample *t*-test with continuity correction, with a significance level threshold of 0.05. Additionally, to assess the differences in the MIC values between the biological replicates of the BFF and BCF isolates, a non-parametric Wilcoxon rank-sum test was chosen to account for unequal sample sizes and a potential non-normal distribution. To evaluate the intra-species MIC variability, a Kruskal–Wallis test was performed for each species separately, considering all replicates, but only species with at least two unique MIC values were included in the analysis. All tests were performed in RStudio 4.2.3^®^ (Posit PBC, Boston, MA, USA) [47,48].

## 3. Results

### 3.1. Biocide Concentrations in the Façades After 1-Year Field Trail

The film preservative concentrations measured of the OIT and TER were 43 ± 8 mg * m^−2^ ± 3% and 296 ± 60 mg * m^−2^ ±11% in the BCFs, which correspond to 14 ± 3% and 56 ± 11% of the initial concentrations, respectively. These initial concentrations were previously reported [19] from the same field trial experiment when using HPLC-UV/VIS detection, ensuring consistency in the reference values. No biocides were detected in the BFFs (excluding residual concentration of BIT), as has also been observed and recently published [19].

### 3.2. Façade Isolate Characterization

**Figure 2:**
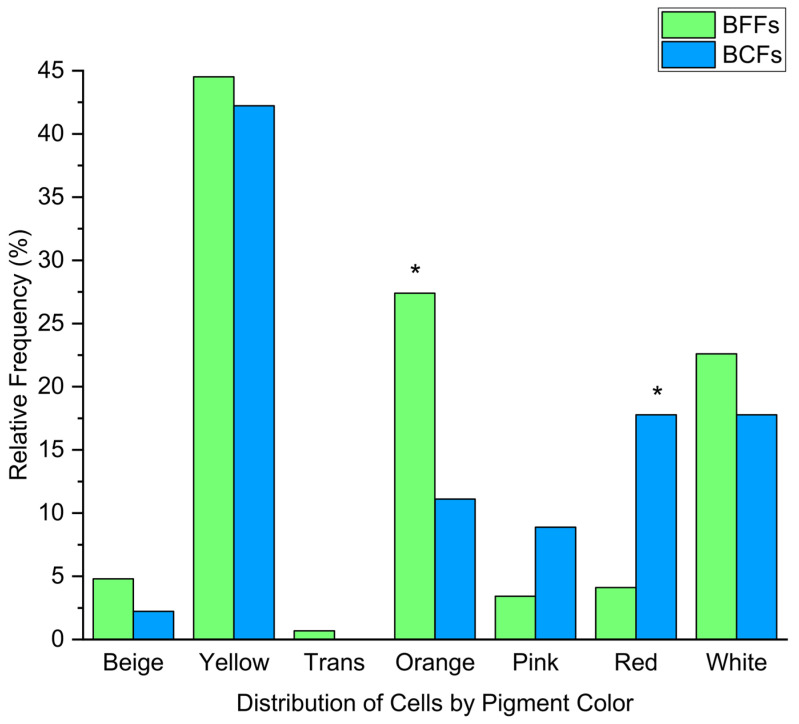
The relative frequency of pigment colorization from the isolates retrieved from the BFFs (*n* = 146 isolates) and BCFs (*n* = 45 isolates). Significant differences (orange: *p* = 0.027, red: *p* = 0.005) are indicated by an asterisk.

In total, 217 bacterial (BFFs: 161 species; BCFs: 56 species) and 26 fungal isolates (BFFs: 22 species; BCFs: 4 species) were identified by Sanger sequencing of the bacterial 16S rRNA gene and fungal ITS gene region, respectively. After excluding the isolates of the genus *Staphylococcus* as potential human contamination, we revealed 146 and 45 bacterial BFF and BCF isolates, respectively (all data, including our isolates of the genus *Staphylococcus*, can be found in the Supplementary Materials). While the BFF isolates were 32.2% Gram-negative, 86.3% catalase positive, and 11.6% oxidase-positive, the BCF isolates non-significantly differed at 35.6% Gram-negative, 82.2% catalase positive, and 6.7% oxidase-positive (Gram-negative: *p* = 0.81; catalase positive: *p* = 0.66; oxidase-positive: *p* = 0.42). The pigment color of all isolates was assessed after receiving a pure culture, as some isolates could not be recovered from the initial culture media. The bacterial BFF isolates were more likely to be orange and beige, while the BCF isolates were more frequently pink and red (Figure 2). Although both the BFFs and BCFs were oriented in the same cardinal direction, the quantity of orange- and red-pigmented bacteria (orange: *p* = 0.027, red: *p* = 0.005), as all façade compounds, was similar between the BCFs and BFFs except for the presence of biocides.

**Figure 3:**
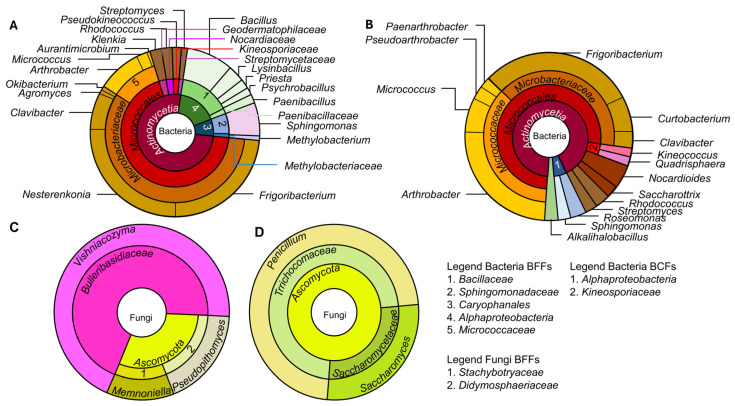
Sunburst plot with the mean abundance of the cultivation-dependent fraction of bacteria (**A**,**B**) and fungi (**C**,**D**) isolated from the BFFs (**A**,**C**) and BCFs (**B**,**D**), all derived from the same south/east orientation.

The bacterial isolates retrieved from the BCFs and BFFs differed significantly in their taxonomic composition (W = 209.5, *p* = 0.046) (Figure 3A,B). The BCF isolates were more dominantly composed of members of the bacterial family *Micrococcaceae* (particularly *Arthrobacter*) and less dominantly of members of *Bacillaceae* compared to the BFF isolates. Additionally, the bacterial families *Geodermatophilaceae*, *Paenibacillaceae*, and *Methylobacteriaceae* were absent in the BCF isolates. Likewise, members of the genus *Nesterenkonia* were not observed on the BCFs. In turn, isolates of the families *Nocardioidaceae* and *Pseudonocardiaceae* were only present on the BCFs. Members of the genera *Arthrobacter*, *Clavibacter*, *Frigoribacterium*, *Micrococcus*, *Rhodococcus*, *Streptomyces*, and *Sphingomonas* were found on both façade types.

The fungal isolates differed at the family level between the BFFs and BCFs and were potentially under-represented. The fungal families *Stachybotryaceae*, *Didymosphaeriaceae*, and *Bulleribasidiaceae* were present in the BFFs, whereas *Saccharomycetaceae* and *Trichocomaceae* were isolated in the BCFs (Figure 3C,D). Although fewer fungal isolates were retrieved compared to published data [7], a clear difference in the taxonomic composition between the BFFs and BCFs was obtained.

### 3.3. MIC of Isolated Strains

**Figure 4:**
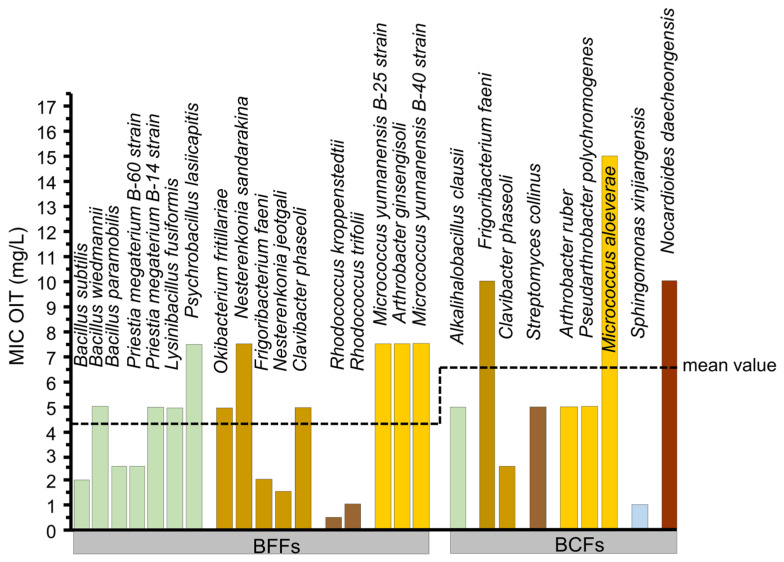
The minimum inhibitory concentrations (MICs) of the octylisothiazolinone (OIT) for bacterial isolates obtained from the BFFs and BCFs, all facing the same south/east orientation. The mean MIC values from the BFF and BCF isolates were non-significantly different (*p* = 0.211). The color code differentiates different taxa at the family level. Bar colors correspond to the taxonomic groups shown in Figure 3A,B, allowing comparison between community composition and resistance patterns.

The mean MIC of the BFF isolates was statistical and the same compared to those of the BCF isolates (BFF mean 4.38 mg * L^−1^; BCF mean 6.5 mg * L^−1^; *p* = 0.21) (Figure 4). The median MIC in the BCF isolates was 5.0 (IQR = 4.75, *n* = 18), whereas in the BFF isolates, the median MIC was 2.5 (IQR = 3.00, *n* = 36). However, a Wilcoxon rank-sum test showed that this difference was not statistically significant (*p* = 0.2999). The intra-species analysis demonstrated that each species was within an acceptable threshold of its MIC variability. The lowest *p*-value was presented by *Priestia megaterium* (*p* = 0.102), which still did not reach statistical significance. Moreover, the MIC range of the BFF isolates (0.5–7.5 mg * L^−1^) was narrower compared that of the BCF isolates (1–15 mg * L^−1^). However, two taxonomic similar BFF isolates, *Priestia megaterium* (strain B-60) and (strain B-14), differed in their susceptibility to OIT by a 2-fold increase (Figure 4), indicating a MIC variability within the same species. Nevertheless, when comparing the *Frigoribacterium faeni* isolates from the BFFs and BCFs, they exhibited a 5-fold difference in MIC against OIT (BFFs: 2 mg * L^−1^ and BCFs: 10 mg * L^−1^). In contrast, *Clavibacter phaseoli* was also isolated from the BCFs and BFFs but showed an inverse relationship with a 2-fold MIC difference (BFFs: 5 mg * L^−1^ and BCFs: 2.5 mg * L^−1^) (Figure 4).

**Figure 5:**
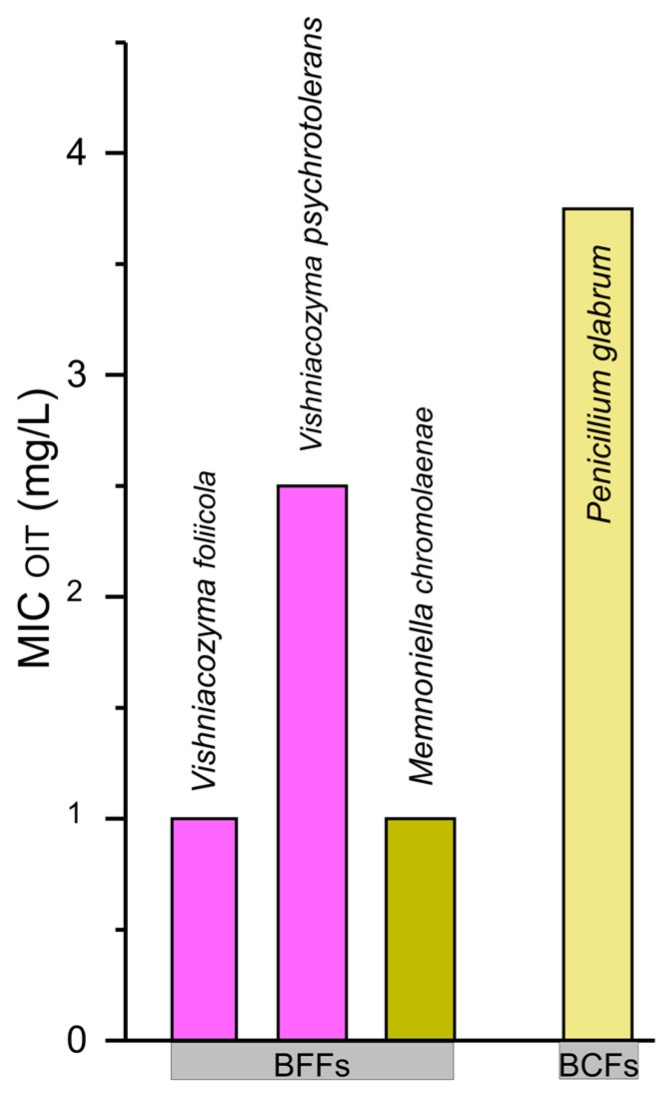
The minimum inhibitory concentrations (MICs) of octylisothiazolinone (OIT) for the fungal isolates retrieved from the BFFs and BCFs, all facing the same south/east orientation. Bar colors correspond to the taxonomic groups shown in Figure 3C,D, allowing comparison between community composition and resistance patterns.

OIT is effective against fungi and is widely regarded as a broad-spectrum biocide with strong antifungal properties. It inhibits a variety of fungi, molds, and yeasts, making it useful in paints, coatings, and other applications where fungal growth control is essential [1,19]. Therefore, we also investigated the MIC against OIT for a few fungal isolates. The fungal MIC values ranged from 1–2.5 mg * L^−1^ in the BFFs and 3.75 mg * L^−1^ in the BCFs (Figure 5).

## 4. Discussion

### 4.1. Biocide Presence Shaped Microbial Community Compositions

Although biocides have been reported to play a significant role in shaping the microbial colonization patterns on façades [49], our results did not show significant shifts in the Gram, oxidase, or catalase status between the BCF and BFF isolates. However, orange pigmentation was significantly more abundant in the BFF isolates compared to the BCF isolates (Figure 2), whereas red pigmentation was significantly more abundant in the BCF isolates. One possible explanation for the higher frequency of red-pigmented isolates in the BCFs is the ecological role of carotenoid and melanin pigments, which protect microorganisms against oxidative stress and toxic compounds [50,51]. Their enrichment under biocidal conditions suggests that pigmentation may support microbial persistence despite chemical control measures, and they may represent an adaptive trait of façade colonizers.

This aligns with previous findings that the presence of biocides can alter microbial community composition, which, in turn, may influence the pigmentation of rock colonizers [52,53]. However, more research is needed to disentangle the relationship between the occurrence and intensity of pigmentation and the presence of biocides. The presence of biocides, along with dirt and other airborne particles on façades, can serve as an organic-based microbial source. Whether these biocides also contribute to the microbial necromass from biocide-susceptible microorganisms remains an ongoing debate [54]. In addition to these organic inputs, primary substrates on facades include sunlight-driven inputs, as well as airborne and façade-based chemolithotrophic substrates [3,54]. The diverse constraints and substrate variations on façades make it challenging to replicate these conditions accurately, including both field trials and, especially, tests under laboratory conditions. Consequently, each cultivation approach only partially mimics the façade environment, making it difficult to establish optimal cultivation substrates and conditions for all the microorganisms present on façades. Although meteorological data (temperature, rainfall, and humidity) were recorded during the exposure period, they were used only to describe environmental conditions as the dataset was too limited for robust statistical correlations with microbial abundance or resistance. Moreover, the BFFs and BCFs were exposed at a single site in Germany with a south-east orientation and scope on biocide presence. The results should, thus, be regarded as site- and climate-specific since microbial colonization is known to vary with geographic location, climate, and façade orientation [55]. Further studies from additional sites are needed to generalize our initial observations, with a particular focus on bacterial isolates. Despite these limitations, our results revealed significant taxonomic differences in the cultivable bacterial fractions of the BCF and BFF isolates (Figure 3). The presence of biocides on façades caused a clear decrease in bacterial abundances (BFFs: 161 species; BCFs: 56 species), which is consistent with an earlier report [55]. Although cultivation-independent methods cannot be directly compared with cultivation-dependent methods, a similar reduction in microbial richness was observed as a decrease in ASV richness from 139 ASVs before the biocide treatment on façades to 21 ASVs afterward [55]. Notably, their study was conducted on surfaces that were first colonized under biocide-free conditions and then, subsequently, treated with a broad spectrum of biocides. In contrast, the facades in our study were colonized in the continuous presence of preservative biocides, reflecting a real-world scenario. Nevertheless, both studies demonstrated the microbial response to biocide exposure. Furthermore, previous studies have obtained *Pseudoarthrobacter* sp. and *Quadrisphaera* sp. after biocidal treatment, which we also exclusively obtained from the BCFs [55]. This suggests that members of both genera are capable of adapting to the presence of biocides [55]. In addition, some of these bacterial and fungal genera retrieved from the BCFs can potentially degrade biocides. For instance, members of the genus *Pseudoarthrobacter* degrade phenanthrene [56] and phenolic compounds [57], which are the backbones of some pesticides, herbicides, and environmental contaminants [56,57]. On the other hand, members of the genus *Quadrisphaera* sp. have shown an increased abundance in heavy metal-polluted arid loess [58], potentially reflecting an adaptation process that enables them to colonize façades treated with biocides. Additionally, bacterial cell extracts from *Clavibacter michiganensisc* [59] and *Arthrobacter nicotinovorans* [60]—both of which we isolated from the BFFs and BCFs—were capable of degrading atrazine, a commonly used herbicide [61]. Many chemotrophic bacteria from the genera *Bacillus*, *Paenibacillus* (isolated from the BFFs), *Arthrobacter*, and *Micrococcus* (isolated from both the BFFs and BCFs) have been frequently isolated from various wall paintings [44], and they are known to promote biodeterioration due to extracellular acid emissions [43]. In addition, *Sphingomonas* (isolated from the BFFs and BCFs) has been repeatedly found on different façades, and stones are described as key drivers in the biodeterioration of stone monuments [62] due to biofilm formation [63] and the production of yellow pigments as a shield against UV radiation [64].

Discoloration, as part of aesthetic biodeterioration, can be caused by several heterotrophs, such as actinomycetes, fungi, and yeasts [11,65]. Ancient building façades were colonized by bacterial community members that play a role in discoloration processes, which include *Micrococcus* (isolated from the BFFs and BCFs), *Bacillus*, and *Streptomyces* (isolated from the BFFs) [66].

Interestingly, we also identified the fungal genus *Penicillium* (isolated from the BCFs), which was found in fungal biofilms associated with surface discoloration, including brownish pigmentation [44]. Moreover, many strains of the genus *Penicillium* can degrade triazine herbicides (such as TER), which potentially contribute to TER reduction [67,68,69]. Moreover, members of the genus *Arthrobacter* sp. isolated from the BCFs and BFFs are described as atrazine (pesticide)-catabolizing bacteria similar to *Penicillium* sp. [59,70,71]. Kumar et al. presented a list of potential pesticide-degrading microorganisms, which includes, among others, *Micrococcus*, *Streptomyces*, *Rhodococcus* (isolated from the BFFs and BCFs), and *Nocardioides* (isolated from the BCFs) [71], indicating that such bacterial functional traits are beneficial on façades. These findings suggest that our isolates could be valuable for future bioremediation applications and could be comparable with the *Phanerochaete chrysosporium* effect on MIT degradation in building environments [72], as well as beyond façade environments [73]. The presence of such biocide degraders can modulate microbial communication systems, interactions, and metabolic pathways within façade biofilm structures, as reported for soil-borne herbicide degraders [74]. Consequently, biocide presence and composition will ultimately impact both biocide-tolerant and biocide-susceptible members of a façade colonizing community. While both OIT and TER are commonly used as film preservatives, our study focused on OIT due to its broader antimicrobial spectrum and environmental availability. TER targets primarily algae, and other photosynthetic organisms were excluded because of the slow pace of colonization and also because of specialized requirements in cultivation and sequencing protocols that were beyond the scope of our study [75,76,77].

### 4.2. Microbial Susceptibility of OIT and the Capabilities to Form Biofilms

The choice of OIT over TER was primarily based on the solubility limitations of TER in MHB. Additionally, TER is known as an algicide that disrupts photosynthesis, and isolated bacterial and fungal strains can be referred to as non-target organisms. However, previous findings have shown both the antifungal and antibacterial properties of TER [78], probably due to the formation of metabolites with changed antimicrobial properties over time [79,80]. The MIC values for OIT did not differ significantly between the isolates retrieved from the BFFs and BCFs, suggesting that biocide exposure did not select for higher biocidal tolerance. The absence of significant intra-species variability underlines the consistency within one species of our isolates. However, the observed differences in community composition between the BFFs and BCFs indicate that the presence of biocides affects colonization patterns. Early colonization may be driven by less susceptible species or by microbes capable of adapting during biocide exposure. Notably, *F. faeni* showed a 5-fold higher MIC from the BCF compared with the BFF isolates (see Figure 4). However, it is important to note that direct comparisons between the two *F. faeni* isolates (from the BFFs and BCFs) were not possible, as their genotypic backgrounds were not analyzed at the genomic level. Such variability in MIC values highlights the ecological relevance of resistance heterogeneity in façade-associated microbial communities. Biocides can drive the adaptation of microbial communities, leading to increased abundance and the emergence of tolerances or resistances in bacterial and fungal strains [81,82,83,84,85]. For instance, the fungi were isolated from cave environments using the OIT disc diffusion method, and it was focused on members of the genus *Penicillium* (BCF isolates, Figure 3 and Figure 5) [33]. However, the reported MICs of the OIT in the same study ranged from 100 mg * L^−1^ to 500 mg * L^−1^ for the *Penicillium* isolates, which is 100-fold higher compared to our study [33]. Other studies have assessed the susceptibility of *Escherchia coli* and *Shizosaccharomyces pombe* to biocides such as BIT, CMIT, and MIT [86], which are in a similar concentration range as in our study (Figure 4). In contrast, the susceptibility of OIT to *Aspergillus niger* and *Saccharomyces cerevisae* [25] is in a 100- to 10-fold lower concentration range than our study, with MIC = 0.05 ± 0.01 mg * L^−1^ and MIC = 0.56 ± 0.10 mg ± * L^−1^, respectively.

Biofilms play a crucial role in the microbial colonization of façades in multiple ways. They offer protection against harsh environmental conditions, such as UV radiation, desiccation, and temperature fluctuations. In addition, biofilms serve as reservoirs for water and nutrients, enabling microbial survival and growth on otherwise nutrient-poor surfaces. They also provide a structured habitat that supports microbial food webs and facilitates genetic exchange through horizontal gene transfer, contributing to microbial adaptation and resilience. Capability in biofilm formation and survival strategies by colonizing organisms is needed to maintain survival on façades. Members of the genera *Sphingomonas*, *Roseomonas* (isolated from the BFFs and BCFs), and *Quadrisphera* (isolated from the BCFs) have been described as the most abundant bacteria involved in biofilm formation before biocidal treatment [55], and they were also present on our façades. In addition, members of the genus *Streptomyces* (isolated from the BFFs and BCFs) are also known to be biofilm formers on striking surfaces [87]. Biofilm formation on façades is shaped by the presence of biocides (e.g., fungicides) that select for insensitive microbial colonizers. Such a presence of biocides promotes intercellular signaling, horizontal gene transfer, and reduced biocide susceptibility [88,89,90]. By protecting tolerant subpopulations and promoting genetic exchange, biofilms also increase the risk of stable, long-term resistance development. This may compromise façade durability and underscores the need for sustainable preservation strategies [88,89,90,91], such as plant-derived biocides [92,93].

Further imaging approaches should resolve if and how such microbes are involved in biofilm formation on real façades, and this study opens this research exploration as façade isolates are now available. Besides their potential contribution to biofilm formation, members of the genus *Streptomyces* have been described as possessing a multidrug-resistant system that enhances efflux pump activity to release antimicrobial agents [94].

## 5. Conclusions

The main objective of this study was to investigate how the application of biocides on façades affects culture-based bacterial and fungal community composition, resistance patterns, and shifts in pigment distribution. We found that biocide application on façades shifted the microbial diversity, leading to significant differences in pigmentation distribution and a decrease in total isolate abundance. Although the median MIC for OIT was slightly higher in the BCF isolates, this difference was not statistically significant. However, the broader MIC range observed in the BCFs may suggest a selective advantage for less susceptible microorganisms or colonization under reduced biocide availability due to local depletion or inactivation. Future studies could incorporate multiple approaches for a more comprehensive evaluation of biocide performance and microbial adaptation, such as monitoring the genotypic and phenotypic characteristics of the isolates growing on the same and other façades.

## Data Availability

Data is included in this publication and additional information will be made available on request.

## Supplementary Materials

The following supporting information can be downloaded at: https://www.mdpi.com/article/10.3390/microorganisms13102284/s1, Table S1: List of isolate information (taxa, color), Gram, Catalase, and oxidase stats.
